# Failure of the glymphatic system as possible link between lumbar spinal stenosis and dementia

**DOI:** 10.1002/alz.13812

**Published:** 2024-04-27

**Authors:** Pasquale Gallina, Francesco Lolli, Berardino Porfirio

**Affiliations:** ^1^ Careggi University Hospital, Neurosurgery Unit Florence Italy; ^2^ Careggi University Hospital, Neurophysiology Unit University of Florence Florence Italy; ^3^ Department of Clinical and Experimental Biomedical Sciences “Mario Serio” University of Florence Florence Italy

Lumbar spinal stenosis (LSS) is a disease of aging.^1^ It involves reduction of the spinal canal with compression of the nerve roots and vascular elements. This mainly degenerative condition results in posture‐related low back and lower extremity pain. Symptoms are relieved by sitting or forward flexion. The cardinal sign of LSS is neurogenic claudication, that is, onset of weakness, tiredness/heaviness of the legs initiated by walking a short distance, which gradually intensifies and obliges patients to stop. Approximately 20% of people over 60 have imaging evidence of LSS. A total of 266 million individuals (3.63%) worldwide were found to have symptomatic LSS or other spinal degenerative conditions (disk degeneration, spondylolisthesis), which will culminate in LSS. These findings understate the true global burden of LSS since quantification in low‐/middle‐income countries is deficient. LSS prevalence is expected to increase with the aging of populations.

LSS is an independent risk factor for dementia.^2^ Over one‐third of patients with LSS had mild cognitive impairment in a Japanese population.^3^ Further studies are needed to assess the generalization of this association.

These findings^2^, ^3^ represent an opportunity to discuss the pathogenesis of dementia in an unexplored model and to underpin the hypothesis that dementia is the final consequence of failure of the glymphatic system (GS) possibly resulting from extra‐intracranial hydrodynamic derangements.^4^

The GS is a brain‐wide fluid transport pathway.^5^ Cerebrospinal fluid (CSF) moves through arteriolar perivascular spaces driven by arterial pulsation, clears interstitial solutes, and leaves the brain, mainly via venular perivascular spaces. If perivascular space flow fails, GS function is deranged and neurodegeneration occurs. An imaging marker of GS flow dysfunction is dilatation of perivascular spaces due to CSF accumulation/stasis.

Reduced glymphatic efflux has been described in normal‐pressure hydrocephalus^6^ and hypothesized in conditions characterized by impairment of cranial CSF outflow by systemic venous hypertension.^4^, ^7^ Similarly, the failure of this veno–CSF–glymphatic fluid connection might explain dementia in LSS due to hypertension of another venous system, the cerebrospinal one. The latter is a valveless, large‐capacity fluid reserve anatomo‐functionally connecting the veins and venous plexuses of the brain and spine.^8^ The vertebral venous plexuses (VVPs) – the Batson plexuses – include the internal and external ones (both coursing longitudinally from the cranial vault to the sacrum). The internal VVPs lie within the spinal canal externally to the dura, while the external VVPs surround the vertebrae. A third type of VVP are the Basi vertebral veins, which run horizontally and lie within the vertebrae. VVPs are connected to the brain at the level of the cranial sinus. Importantly, VVPs have a bidirectional flow. They represent the major outflow track of cerebral venous drainage in the erect position; outflow through the internal jugular veins is absent/negligible in this position. These hydraulic characteristics of VVPs are key to our speculation about the pathogenetic link between LSS and dementia.

Multilevel LSS can lead to venous congestion/stasis in the intervening segments.^9^ In this anatomical condition, the arterioles will continue to feed these segments at higher arterial pressure, but the impaired drainage reduces blood flow with an increase of venous pressure.^9^ These venous derangements are augmented by spinal extension and rotation, which further reduce the cross‐sectional area of the central spinal canal and neural foramina, triggering LSS symptoms. Moreover, during walking, venous return is increased by action of the lower limbs and accompanied by engorgement of the pelvic veins and VVP.^9^ Dilatation of internal VVPs results in a further narrowing of the spinal canal on a hemodynamic basis, possibly explaining the neurogenic claudication.^9^ This self‐sustaining mechanism will intensify vertebral venous hypertension.^1^

Dementia in multilevel LSS might be related to reduced cranial venous and CSF outflows, determining venous perivascular space engorgement and GS malfunctioning. A possible step‐by‐step demonstration of our hypothesis relies on assessing the association of LSS and dementia in one‐level versus multilevel stenosis. In subjects with LSS who do not present known^5^ or hypothesized^4^, ^7^ causes of GS flow failure, a higher prevalence of enlarged perivascular spaces, compared to healthy subjects, might support GS derangement as a cause of the dementia, tracing the path for autoptic examination in search of GS pathology.^10^

To support this argument, in obesity, whose relationship with dementia has been reported,^11^ an increase in body size is associated with greater burden of dilated perivascular spaces, compared to that observed in subjects experiencing body size decrease.^12^ Since the cerebrospinal venous system is in open communication with the intrathoracic and intra‐abdominal veins, the failure of the GS, likely responsible for dementia in obese subjects, might rely on mechanisms similar to those advocated in LSS.

If further studies demonstrate the connection between cognitive decline and LSS by the involvement of the cerebrospinal venous system, then walking, one of the most frequently encouraged activities for wellbeing, should be limited in patients with asymptomatic multilevel LSS, and dementia could ultimately be prevented through surgical decompression of the lumbar spine.

## AUTHOR CONTRIBUTIONS

**Pasquale Gallina**: Study conception and drafting the article. **Francesco Lolli**: Acquisition of data and revising paper critically for important intellectual content. **Berardino Porfirio**: Drafting article and revising it critically for important intellectual content. All authors gave approval of the version to be submitted.

## CONFLICT OF INTEREST STATEMENT

The authors have no competing interests to declare. Author disclosures are available in the supporting information.

## Supporting information

Supporting Information
